# Clinical and Echocardiographic Outcomes of Transcatheter Tricuspid Valve Interventions: A Systematic Review and Meta-Analysis

**DOI:** 10.3389/fcvm.2022.919395

**Published:** 2022-07-11

**Authors:** Anna Sannino, Federica Ilardi, Rebecca T. Hahn, Patrizio Lancellotti, Philipp Lurz, Robert L. Smith, Giovanni Esposito, Paul A. Grayburn

**Affiliations:** ^1^The Heart Hospital Baylor Plano, Plano, TX, United States; ^2^Department of Advanced Biomedical Sciences, University Federico II, Naples, Italy; ^3^Mediterranea Cardiocentro, Naples, Italy; ^4^Division of Cardiology, Columbia University Irving Medical Center, New York, NY, United States; ^5^Department of Cardiology and Radiology, GIGA Cardiovascular Sciences, CHU SartTilman, University of Liège Hospital, Liège, Belgium; ^6^Gruppo Villa Maria Care and Research, Lugo, Italy; ^7^Department of Internal Medicine/Cardiology, Heart Center Leipzig at University of Leipzig and Leipzig Heart Institute, Leipzig, Germany

**Keywords:** tricuspid regurgitation (TR), transcatheter tricuspid intervention, Echocardiography, survival, outcomes

## Abstract

**Background:**

Medically managed tricuspid regurgitation (TR) has detrimental outcomes. Transcatheter tricuspid valve interventions (TTVIs) represent an alternative to surgery in high-risk patients; however, only early experiences exist.

**Aim:**

The aim of this study was to analyze the clinical and echocardiographic outcomes of TTVI.

**Methods:**

MEDLINE, ISI Web of Science, and SCOPUS databases were searched for studies published up to June 2021. Studies reporting data on outcome post-TTVIs were included. This study was designed according to Preferred Reporting Items for Systematic Reviews and Meta-Analyses (PRISMA) requirements. The primary endpoint was all-cause mortality at 30-day and 1-year post-TTVI.

**Results:**

Out of 2,718 studies, 27 were included. Notably, 30-day and 1-year all-cause mortalities were 5% (95% confidence interval [CI]: 4–8%, *p* < 0.001) and 25% (95% CI: 12–45%, *p* = 0.016). Procedural success was associated with a 58% risk reduction in 1-year mortality vs. lack thereof (odds ratio 0.42, 95% CI: 0.27–0.66, *p* < 0.001). TTVI is associated with a significant reduction in TR severity (TR EROA, mean difference [MD] 0.31 cm^2^; 95% CI: 0.23–0.39 cm^2^, *p* < 0.001; regurgitant volume, MD 23.54 ml; 95% CI: 17.4–29.68 ml, *p* = 0.03) and increase in forward stroke volume (FSV, MD 3.98 ml; 95% CI: 0.11–7.86 ml, *p* = 0.04).

**Conclusion:**

TTVI significantly reduces TR severity and increases FSV and is associated with improved survival at 1 year compared with patients without procedural success. Long-term outcomes compared with medical therapy await the results of ongoing pivotal trials; nonetheless, TTVIs appear to be a promising alternative to surgery for TR.

## Introduction

Tricuspid regurgitation (TR) represents an important healthcare burden, which has often been neglected or undertreated in the past (1). Recent prevalence studies suggest that in >90% of the cases, TR has a functional etiology secondary to left heart disease, pulmonary hypertension primary right ventricular (RV) dilation, and/or dysfunction or right atrial/annular dilation (2). The latter etiology is now referred to as atrial functional TR commonly due to long-standing atrial fibrillation (3). In the remaining cases, TR is considered primary (including prolapse, flail, carcinoid, or other inflammatory diseases as well as pacemaker lead impingement/perforation or adherence to TV leaflets) (1). TR is associated with detrimental outcomes, independently of RV dysfunction or pulmonary hypertension (4). This observation has led to the expansion and reinforcement of the indications for TV intervention in the newest editions of the guidelines (5); however, isolated surgical TV interventions have been associated with ~8–10% in-hospital mortality (6, 7) fostering intense interest in transcatheter tricuspid valve interventions (TTVI) (8). Depending on the anatomic target, TTVI can be categorized as follows: (1) leaflet grasping devices (edge-to-edge repair) or spacers to restore leaflet coaptation; (2) direct or indirect tricuspid restrictive annuloplasty; (3) orthotopic transcatheter TV replacement (TTVR); and (4) heterotopic transcatheter heart valve implantation (caval implantation devices – CAVI). Several studies have been published on the feasibility of the above-mentioned techniques; however, large observations/randomized clinical trials are still lacking. In this systematic review and meta-analysis, we offered an overview of all the available evidence on the topic, reporting analytic data on relevant clinical and echocardiographic outcomes.

## Methods

### Literature Search and Study Selection

This study was designed according to Preferred Reporting Items for Systematic Reviews and Meta-Analyses (PRISMA) requirements (9). MEDLINE, ISI Web of Science, and SCOPUS databases were searched for studies published up to 15 June 2021. Studies were identified using the major medical subject heading “tricuspid regurgitation AND transcatheter OR percutaneous AND survival OR mortality OR outcome.” English was set as a language restriction. Two authors (AS and FI) independently examined the title and abstract of citations. The full texts of potentially eligible trials were obtained, and disagreements were resolved by discussion. To look for additional relevant studies, the full texts and bibliography of all potential articles were also retrieved in detail. Abstract, meeting proceedings, and personal communications were not used for the purpose of this study.

### Eligibility Criteria

Studies were included if they reported data on outcomes of any kind of TTVI. Studies were excluded if any of the following criteria applied: (1) duplicate or overlapping publication data; (2) lack of outcome data; and (3) the outcome of interest was not clearly reported or was impossible to extract or calculate from the published results. Follow-up length was not set as a restriction.

### Data Extraction

Two reviewers independently screened articles for the fulfillment of inclusion criteria (AS and FI). Baseline characteristics and clinical and echocardiographic outcomes were abstracted. Reviewers compared selected trials, and discrepancies were resolved by consensus.

### Endpoints and Definitions

The primary endpoint (or outcome) of this study was to evaluate the overall incidence of all-cause mortality at 30 days and 1 year after TTVI. We additionally evaluated the incidence of all-cause mortality in patients with successful vs. unsuccessful TTVI procedures. Procedural success was defined as the patient alive at the end of the procedure, with the device successfully implanted and the delivery system retrieved, with a residual TR grade of ≤ 2+ (10). We additionally evaluated the change in the following echocardiographic outcomes from baseline to 30-day post-TTVI: effective regurgitant orifice area (EROA), regurgitant volume, left ventricle forward stroke volume (FSV), fractional area change (FAC), tricuspid annular plane systolic excursion (TAPSE), TV annular diameter, RV basal diameter, right atrial volume, TV mean gradient, and pulmonary artery systolic pressure (PASP). As secondary endpoints, we analyzed (1) cardiovascular mortality, (2) rehospitalization for heart failure; and (3) 6-month mortality.

### Quality Assessment

The risk of bias for each included study was assessed using the Newcastle-Ottawa quality assessment scale, as previously described (11). This scale allows the assessment of the internal validity of cohort studies included in the meta-analysis on the basis of three main items: (1) selection (adequate selection and definition of groups); (2) comparability (comparability of two groups for a selected variable and comparability for other variables); and (3) outcome (modality of assessment, enough length of follow-up, and adequacy of follow-up). Based on the above criteria, studies with 4 stars for selection, 2 for comparability, and 3 for outcome were defined at low risk of bias. Studies with 2 or 3 stars for selection, 1 for comparability, and 2 for outcome were defined at medium risk. Any study with a score of 1 for selection or outcome ascertainment, or 0 for any of the three domains, was deemed at high risk of bias.

### Statistical Analysis

Two investigators independently extracted for each study the most comprehensively adjusted/unadjusted odds ratio (OR) and their 95% confidence intervals (95% CIs) as well as means ± standard deviations. Estimates of effect were calculated with a random-effects model and expressed as OR or event rates. Statistical significance was set at *p* ≤ 0.05 (2-tailed). Heterogeneity was assessed by a Q-statistic and *I*^2^ test. Significant heterogeneity was considered present for *p*-values <0.10 or an *I*^2^ > 50%. Meta-regression analysis was performed to assess the potentially important covariates that might exert a substantial impact on between-study heterogeneity (significance at *p* ≤ 0.05) (12). A fixed-effect model was used to confirm the results in case of significant heterogeneity (11).

Publication bias was assessed using funnel plots, and when a significant publication bias was found, it was further explored using the Egger's test, consisting in a linear regression of the intervention effect estimates on their standard errors, weighting by 1/(variance of the intervention effect estimate).

All data analyses were performed using Prometa Software version 2 and Reviewer Manager (RevMan, version 5.2) (11).

## Results

### Identification of Studies

The database search yielded 2,718 studies, of which 39 were retrieved for more detailed evaluation and 27 were included in this systematic review analysis, with a total of 1,216 patients undergoing TTVI (13–39). The process of study selection is summarized in Supplementary Figure 1. All studies were nonrandomized, interventional, and prospective, with follow-up time ranging from 4 to 139 weeks. Different devices were used in the studies, including Cardioband (Edwards Lifesciences, Irvine, California; *n* = 2); Evoque (Edwards Lifesciences; *n* = 1); FORMA (Edwards Lifesciences; *n* = 3); GATE (NaviGate Cardiac Structures, Inc., Lake Forest, California; *n* = 1); MitraClip (Abbott Vascular, Chicago, Illinois; *n* = 12); PASCAL (Edwards Lifesciences; *n* =5); Trialign (Mitralign, Tewksbury, Massachusetts; *n* = 1), and TriClip (Abbott Vascular; *n* = 1). Caval valve implantation (CAVI) was performed in 2 studies, using Edwards Sapien XT or Sapien 3 (Edwards Lifesciences; *n* = 2) and TricValve (P&F, Vienna, Austria; *n* = 1).

### Baseline Characteristics

Patients' mean age was 76.6 years, predominantly women (60.5%), with an 88.2% New York Heart Association (NYHA) functional classes III-IV. Other relevant baseline characteristics are reported in Table 1.

**Table 1:** Baseline clinical characteristics of the included studies.

**Study**			**Year**	**N**	**Male gender (%)**	**Follow up** **(weeks)**	**Mean age (years)**	**BMI** **(kg/m^**2**^)**	**NYHA class III-IV (%)**	**HTN** **(%)**	**Atrial fibrillation (%)**	**Previous CABG** **(%)**	**Previous cardiac surgery (%)**	**Previous mitral valve surgery (%)**			
**Ali et al**. **(****13****)**			2020	40	45	4	75.1 ± 5	25.8 ± 2.2	97.5	65	92.5	22.5	N/A	15			
**Besler et al**. **(****15****)**			2018	43	39	26 (13-35)	78.0 (74.0-83.0)	25.7 (22.9-29.3)	91	N/A	N/A	19	N/A	N/A			
**Braun et al**. **(****16****)**			2018	69	N/A	4	78 ± 11	N/A	100	N/A	N/A	N/A	N/A	N/A			
**Braun et al**. **(****17****)**			2019	31	N/A	4	77 ± 5	N/A	90	N/A	N/A	N/A	N/A	N/A			
**Cai et al**. **(****18****)**			2020	53	41.5	60.8	74.8 ± 11.1	25.5 ± 6.3	93.5	67.9	88.7	20.8	18.9	N/A			
**Camplelo-Parada et al**. **(****19****)**			2015	7	57	4	76 ± 13	N/A	86	N/A	71	N/A	71	29			
**Davidson et al**. **(****20****)**			2021	30	20	4	77 ± 8	N/A	70	62.1	96.7	3.3	N/A	26.7			
**Dreger et al**. **(****21****)**			2020	14	14	42	77 (68.2-82.0)	25.5 ± 4.6	86	N/A	N/A	N/A	21	N/A			
**Fam et al**. **(****22****)**			2019	28	46	4	78 ± 6	N/A	100	N/A	93	18	N/A	N/A			
**Fam et al**. **(****23****)**			2021	25	12	4	76 ± 3	N/A	88	68	84	20	44	24			
**Hahn et al**. **(****24****)**			2017	15	13.3	4	73.6 ± 6.6	N/A	66.7	80	66.7	26.7	60	60			
**Hahn et al**. **(****25****)**			2020	30	44	18 ± 12	78 (70-80)	N/A	86	70	90	33	40	N/A			
**Kitamura et al.(** **26** **)**			2021	30	43	52	77 ± 6	N/A	90	N/A	93	10	N/A	N/A			
**Kodali et al**. **(****27****)**			2021	34	47.1	4	76.3 ± 10.4	N/A	79.4	94.1	88.2	29.4	N/A	20.6			
**Lurz et al**. **(****29****)**			2018	11	73	4	76.9 ± 5.4	N/A	82	91	100	18	N/A	N/A			
**Lauten et al**. **(****28****)**			2018	25	48	52	73.9 ± 7.6	N/A	100	92	N/A	N/A	76	N/A			
**Mehr et al**. **(****30****)**			2019	249	48.6	41 (20-56)	77 ± 9	25.7 ± 4.9	95.6	N/A	73.8	N/A	10.8	N/A			
**Nickening et al**. **(****31****)**			2017	42	45	2 ± 2.5	76.5 ± 9.4	N/A	90	79	86	N/A	43	N/A			
**Nickening et al**. **(****32****)**			2019	85	34	26	77.8 ± 7.9	N/A	75	86	92	N/A	N/A	17.6			
**Nickening et al**. **(****33****)**			2019	30	26.7	26	75.2 ± 6.6	N/A	83.3	80	93.3	23.3	13.3	N/A			
**Orban et al**. **(****34****)**			2020	119	49	51 (28-58)	75.2 ± 10.8	N/A	92	N/A	87	N/A	21	N/A			
**Perlman et al**. **(****35****)**			2018	29	34	4	75.9 ± 8.2	N/A	86	N/A	83	31	48	N/A			
**Perlman et al**. **(****36****)**			2017	18	28	52	76.0 ± 9.7	27.2 ± 5.7	94	89	89	N/A	72	33			
**Rommel et al**. **(****37****)**			2019	29	55	26	78.4 ± 4.0	26.4 ± 4.3	79	97	93	21	21	N/A			
**Ruf et al**. **(****38****)**			2021	50	42	4	80 (78–83)	N/A	98	78	86	N/A	N/A	2			
**Sugiura et al**. **(****39****)**			2020	80	42	13	78 ± 7	25.6 (21.8, 27.4)	93	85	94	N/A	64	N/A			
**Previous aortic valve surgery** **(%)**	**Previous PMK lead (%)**	**LVEF** **(%)**	**LVEDV (mL)**	**Forward SV** **(mL)**	**RV FAC (%)**	**RV diameter (basal, mm)**	**RVEF (%)**	**TAPSE** **(mm)**	**TV mean gradient (mmHg, mean)**	**Severe TR** **(%)**	**PASP (mmHg)**	**RA Volume** **(ml)**	**Tricuspid annulus diameter (mm)**	**Concomitant MR treatment (%)**	**TR EROA (cm** ^**2**^ **)**	**TR regurgitant volume** **(ml)**
17.5	20	47.7 ± 6.3	N/A	53.7 ± 9.5	33.6 ± 4.5	49.5 ± 3.9	N/A	17.3 ± 2.0	N/A	100	N/A	146.6 ± 50.2	47.6 ± 2.9	52.5	0.72 ± 0.12	58.8 ± 7.2
N/A	26	57.0 (45.0-63.0)	N/A	N/A	41.0 (30.0-48.3)	N/A	N/A	15.3 ± 4.8	N/A	100	N/A	N/A	49.7 ± 6.5	0	0.50 (0.40-0.80)	N/A
N/A	N/A	N/A	N/A	N/A	N/A	N/A	N/A	16.1 ± 4.5	N/A	100	N/A	N/A	46 ± 6	61	N/A	N/A
N/A	N/A	N/A	N/A	N/A	N/A	N/A	N/A	17 ± 5	1.1 ± 0.5	92	N/A	N/A	51 ± 9	45	N/A	N/A
N/A	26.4	49.7 ± 16.6	101.8 ± 44	48.8 ± 14.5	N/A	49.4 ± 7.7	N/A	15.6 ± 3.4	N/A	100	N/A	N/A	N/A	N/A	N/A	N/A
29	N/A	56 ± 5	N/A	N/A	N/A	47 ± 7	N/A	16.5 ± 4.2	N/A	100	69.7 ± 6.0	N/A	N/A	N/A	N/A	N/A
16.7	23.3	58.6 ± 5.8	N/A	63.4 ± 16.8	41.6 ± 5.2	5.6 ± 0.6	N/A	N/A	N/A	100	37.8 ± 10.9	134.6 ± 41.6	N/A	N/A	0.84 ± 0.39	N/A
N/A	N/A	56.4 ± 6.4	N/A	N/A	N/A	49.0 ± 6.6	N/A	16.1 ± 5.2	N/A	100	39.0 (33.5-55.5)	N/A	N/A	0	1.23 ± 0.6	68.7 ± 24.6
N/A	3	58.5 ± 6.2	N/A	N/A	N/A	N/A	N/A	15.7 ± 3.3	N/A	100	N/A	N/A	49.5 ± 8	0	1.3 ± 2.4	57.7 ± 16.6
28	36	58.3 ± 3.6	N/A	N/A	37.6 ± 5.1	50.7 ± 3.1	49.2 ± 3.4	15.6 ± 2.5	N/A	100	N/A	N/A	44.7 ± 7.1	0	0.86 ± 0.21	60.2 ± 8
0	0	59.9 ± 11.5	N/A	63.6 ± 17.9	N/A	N/A	N/A	16 ± 4	N/A	N/A	43.6± 9.3	N/A	40 ± 5	0	0.51 ± 0.16	86 ± 21.3
N/A	30	55 (46-60)	N/A	56 (42-65)	N/A	N/A	N/A	14 (12–18)	N/A	93	38 (30-55)	N/A	49 (44-50)	N/A	0.75 (0.7–1.1)	N/A
N/A	3	59 ± 8	N/A	N/A	N/A	44 ± 9	N/A	16.2 ± 3.5	1.0 ± 0.1	100	N/A	N/A	49 ± 10	N/A	N/A	N/A
14.7	11.8	57.4 ± 7.0	N/A	63.9 ± 15.8	38.4 ± 9.0	N/A	N/A	15.3± 4.7	N/A	97	N/A	162.4± 104.8	46.1 ± 7.7	N/A	0.71± 0.33	47.4± 22.5
N/A	9	56 ± 12	N/A	N/A	N/A	41 ± 8	N/A	16 ± 3	N/A	100	36 ± 13	80 ± 30	54± 5	0	0.5 ± 0.4	50 ± 23
N/A	36	51 ± 15	N/A	N/A	N/A	N/A	N/A	16.5 ± 4.1	N/A	100	41.0 ± 13.9	N/A	51 ± 6.7	0	N/A	N/A
N/A	29.7	49 ± 14	N/A	N/A	N/A	N/A	N/A	15.8 ± 4.3	N/A	96.8	43.6 ± 16	106.5 ± 74.6	47 ± 7.6	51.8	0.70 ± 0.53	N/A
N/A	26	50.6 ± 11.4	99.8 ± 56.4	N/A	36.8 ± 12.1	N/A	N/A	16.6 ± 5.2	N/A	86	40.4 ± 14.6	131.2 ± 76.9	43.2 ± 7.6	0	0.8 ± 0.4	59.9 ± 18.4
11	14	59 ± 8	N/A	61.02 ± 14.79	35.83 ± 7.39	52.7 ± 6.7	N/A	14.4 ± 3.1	1.2 ± 0.6	94	38.9 ± 16.0	128.04 ± 53.88	43.3 ± 5.9	0	0.65 ± 0.29	51.63 ±18.65
N/A	13.3	57.2 ± 10.5	N/A	52.1 ± 19.7	N/A	N/A	N/A	N/A	N/A	71	35.8 ± 10.6	N/A	42.2 ± 0.5	N/A	0.79 ± 0.51	79.4 ± 29.6
N/A	27	53.3 ± 12.9	N/A	N/A	38.7 ± 9.9	N/A	40.5	16.4 ± 5.3	N/A	100	43.4 ± 14.2	N/A	46.4 ± 7.6	0	0.61 ± 0.37	N/A
N/A	24	55.9 ± 13.8	N/A	N/A	N/A	59 ± 9	N/A	14 ± 4	N/A	100	N/A	N/A	44 ± 7	0	1.1 ± 0.6	N/A
22	17	59 ± 9	N/A	N/A	N/A	54.0 ± 5.3	N/A	14.7 ± 5.4	N/A	94	43 ± 13	143 ± 59	45.7 ± 4.8	0	1.03± 0.61	N/A
N/A	35	52.0 ± 12.6	N/A	N/A	39.7 ± 8.8	N/A	N/A	16.1 ± 4.8	N/A	100	49.8 ± 14.7	N/A	N/A	N/A	0.6 ± 0.3	51.1 ± 16.5
0	20	55.49 (54.65–59.61)	64.9 (50.8-88.1)	N/A	32.65 (24.78–37.78)	54.0 (49.2–59.1)	N/A	15.5 (10.0–18.0)	1.3 ± 0.9	86	N/A	144.05 (112.48–226.65)	N/A	N/A	N/A	N/A
N/A	31	56.9 (52.8, 62.0)	N/A	N/A	N/A	46.0 (31.0, 53.0)	N/A	17.0 (14.0, 20.0)	N/A	100	N/A	N/A	45.0 (40.0, 52.0)	N/A	0.52 (36.8, 75.8)	50.0 (40.6, 61.0)

*Values are means ± SD, median (IQR), n(%) as appropriate. BMI, body mass index; HTN, hypertension; NYHA, New York Heart Association; CABG, coronary artery by-pass graft; PMK, pacemaker; LVEF, left ventricle ejection fraction; RV FAC, right ventricle fractional area change; RVEF, right ventricle ejection fraction; TAPSE, tricuspid annular plane systolic excursion; TR, tricuspid regurgitation; PASP, pulmonary artery systolic pressure; RA, right atrium; MR, mitral regurgitation; EROA, effective regurgitant orifice area; FVR, forward stroke volume; LVEDV, left ventricle end-diastolic volume*.

### Clinical Outcomes

**Figure 1:**
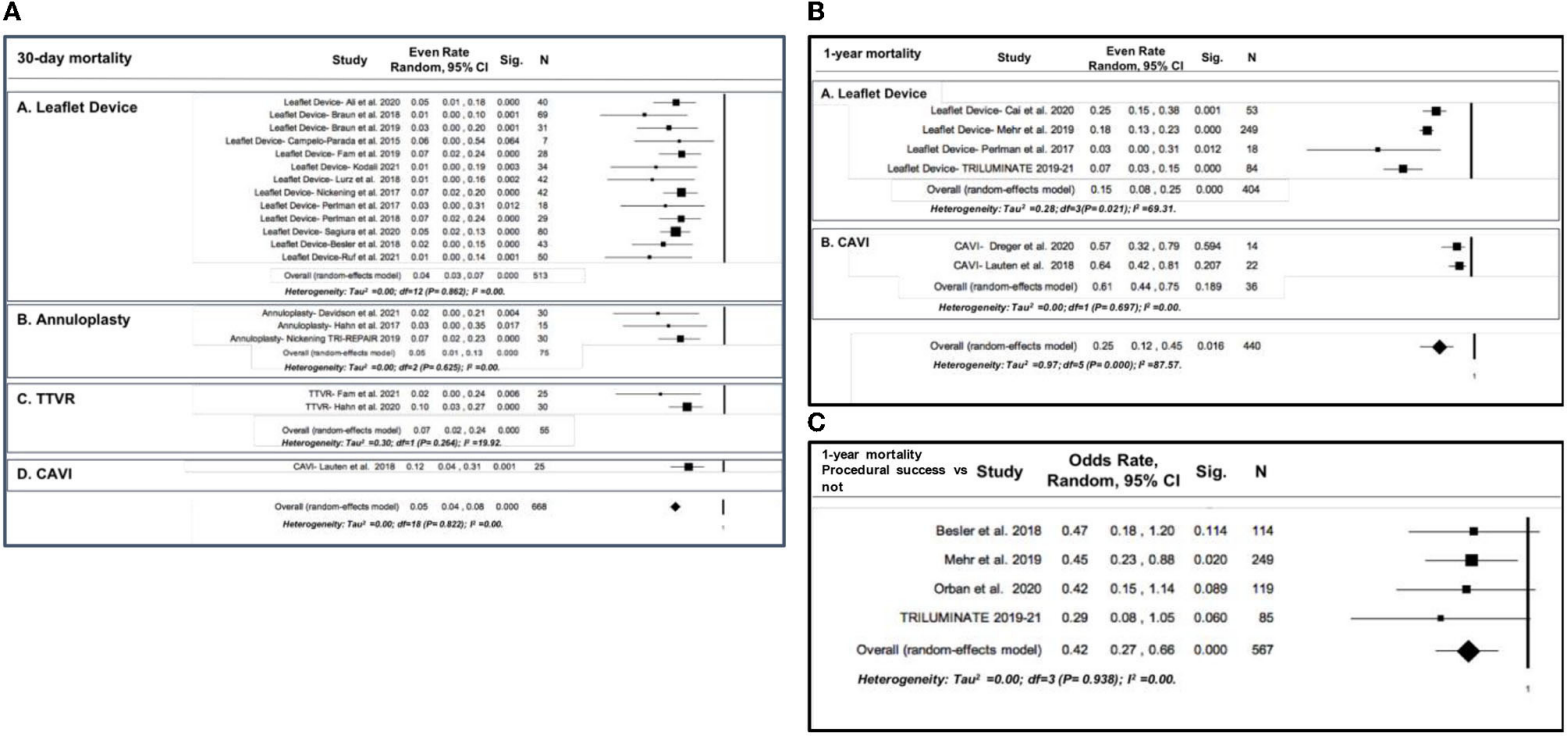
Forest plot for the incidence of all-cause mortality after transcatheter tricuspid valve intervention (TTVI): **(A)** 30-day all-cause mortality, with subgroup analysis by device type; **(B)** 1-year all-cause mortality, with subgroup analysis by device type; **(C)** 1-year all-cause mortality comparison between procedural success vs. procedural unsuccess).

The overall incidence of 30-day all-cause mortality was 5% (21 studies; 95% CI: 4% to 8%, *p* < 0.001, *I*^2^ = 0.00%) (Figure 1A). Subgroup analysis showed a significantly higher mortality rate in the CAVI subgroup vs. leaflet devices, annuloplasty, and TTVR devices (12%, 95% CI: 4 to 31%, *p* = 0.001, *I*^2^ = 0.00 vs. 4%, 95% CI: 3 to 7%, *p* < 0.001, *I*^2^ = 0.00%, 5% 95% CI: 1 to 13%, *p* < 0.001, *I*^2^ = 0.00%, 7% 95% CI: 2 to 24%, *p* < 0.001, *I*^2^ = 19.92%, respectively; test for subgroup difference *p* < 0.0001) (Figure 1A).

At 1 year, the overall mortality rate was 25% (6 studies; 95% CI: 12% to 45%, *p* = 0.016, *I*^2^ = 82.57%) (Figure 1B). Subgroup analysis revealed a significantly higher mortality rate in the CAVI device subgroup vs. leaflet devices (61%, 95% CI: 44 to 75%, *p* = 0.697, *I*^2^ =0.00 vs. 15%, 95% CI: 8 to 25%, *p* < 0.001, *I*^2^ = 69.31%) (Figure 1B). The incidence of death was significantly reduced in those in which procedural success of TTVR was achieved compared with the patients without procedural success (OR 0.42, 95% CI: 0.27 to 0.66, *p* < 0.001, *I*^2^ = 0.00%) (Figure 1C).

When sensitivity analysis was performed by removing the CAVI studies, the overall 30-day (18 studies; 5%, 95% CI: 3 to 6%, *p* < 0.001, *I*^2^ = 0.00%) and 1-year mortality rates (5 studies; 17%, 95% CI: 11 to 25%, *p* < 0.001, *I*^2^ = 68.32%) were slightly but not significantly modified (Supplementary Figures 2A,B).

The overall incidence of cardiovascular mortality was estimated at 10% (95% CI: 3 to 30%, *p* = 0.0001, *I*^2^ = 68.8%) (Supplementary Figure 3A), with a rate of rehospitalization for heart failure of 25% (95% CI: 14 to 42%, *p* < 0.001, *I*^2^ = 55.42%; Supplementary Figure 3B). In the studies assessing 6-month follow-up, the incidence of all-cause mortality was estimated at 9% (4 studies, 95% CI: 4 to 20%, *p* < 0.001, *I*^2^ = 64.5%) (Supplementary Figure 3C). Results were confirmed using a sensitivity analysis; removing one study at a time did not determine in changes in any of the explored outcomes (data not shown).

### Echocardiographic Outcomes

**Figure 2:**
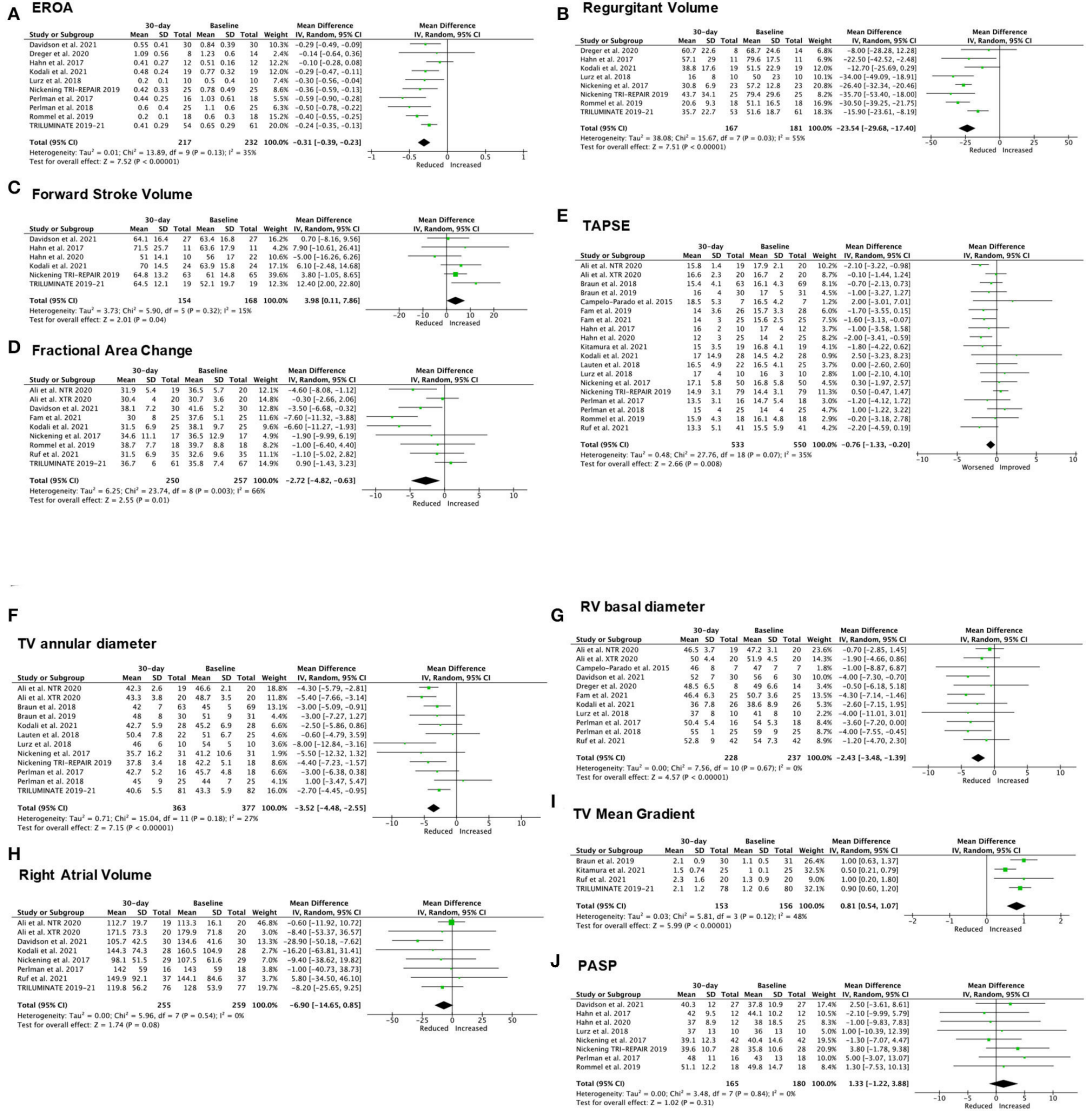
Forest plot for the evolution of echocardiographic outcomes at 30-day post-TTVI. **(A)** Effective regurgitant orifice area (EROA). **(B)** Regurgitant volume. **(C)** Forward stroke volume. **(D)** Fractional area change. **(E)** Tricuspid annular plane systolic excursion (TAPSE). **(F)** TV annulus diameter. **(G)** Right ventricular (RV) basal diameter. **(H)** Right atrial volume. **(I)** TV mean gradient. **(J)** Pulmonary Artery Systolic Pressure (PASP).

Quantitative measurement of TR significantly decreased 30-day after TTVI, including a reduction in EROA (mean difference [MD] −0.31 cm^2^; 95% CI: −0.39 to −0.23cm^2^, *p* < 0.001, *I*^2^ = 35%, Figure 2A) and regurgitant volume (MD −23.54 ml; 95% CI: −29.68 to −17.4 ml, *p* = 0.03, *I*^2^ = 55% Figure 2B), paired with a significant increase in FSV (MD 3.98 ml; 95% CI: 0.11 to 7.86 ml, *p* = 0.04, *I*^2^ = 15% Figure 2C). Conversely, RV function showed a slight, but statistically significant worsening after TTVI, measured as FAC (MD −2.72%, 95% CI: −4.82 to −0.63%, *p* = 0.01, *I*^2^ = 66%, Figure 2D) and TAPSE (MD −0.76 mm, 95% CI: −1.33 to −0.20 mm, *p* = 0.008, *I*^2^ = 35%, Figure 2E). A significant improvement of right heart size was observed at 30-day follow-up, described by a reduced TV annulus diameter (MD −3.52 mm; 95% CI: −4.48 to −2.55 mm, *p* < 0.001, *I*^2^ = 27%, Figure 2F) and RV basal diameter (MD −2.43 mm; 95% CI: −3.48 to −1.39 mm, *p* < 0.001, *I*^2^ = 0%; Figure 2G), while right atrial volume did not change significantly (MD −6.90 ml; 95% CI: −14.65 to 0.85 ml, *p* = 0.08, *I*^2^ = 0%, Figure 2H). Finally, TTVI was also associated with a slight but significant increase in transvalvular gradient (MD 0.81 mmHg; 95% CI: 0.54 to 1.07 mmHg, *p* < 0.001, *I*^2^ =48%; Figure 2I). Overall, no significant variation of PASP was observed post-TTVI (MD 1.33 mmHg, 95% CI: −1.22 to 3.88 mmHg, *p* = 0.31, *I*^2^ = 0%; Figure 2J).

### Study Quality Assessment and Heterogeneity

Heterogeneity assesses whether observed differences in results arise by chance alone. To assess the impact of study quality (bias) on heterogeneity, we applied the Newcastle-Ottawa quality assessment scale to the primary studies included in the meta-analysis. All included studies fell into the categories of “low” or “medium” risk of bias (Supplementary Table 1).

### Meta-Regression and Publication Bias

To explore the potential impact of effect size modifiers on 30-day and 1-year all-cause mortality, we performed a meta-regression analysis of the baseline characteristics of the included studies. At 1 year, a significant relation was found between TAPSE, tricuspid annulus diameter, and mortality (Supplementary Table 2 and Supplementary Figure 4).

The funnel plots did not show any significant publication bias for all the performed analyses (Supplementary Figure 5).

## Discussion

**Figure 3:**
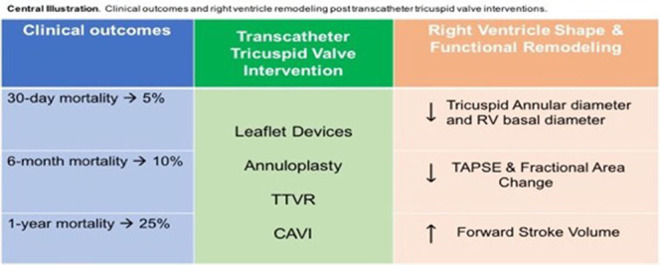
Central Illustration. Clinical outcomes and right ventricle remodeling post transcatheter tricuspid valve interventions.

To the best of our knowledge, this is the first meta-analysis comprehensively assessing the clinical and echocardiographic outcomes of TTVI. The main results of this study can be summarized as follows: (1) overall mortality rates at 30 days, 6 months, and 1 year are 5, 10, and 25%, respectively. (2) TTVI procedural success is associated with a 58% risk reduction in 1-year all-cause mortality compared with the absence of procedural success; (3) TTVI is associated with a significant reduction in TR severity measured as EROA and regurgitant volume, with a contextual increase in FSV despite a reduction in RV function (TAPSE and FAC). RV size improved at 30 days after TTVI (RV basal diameter and TV annulus diameter); right atrial volume change was not statistically significant. Finally, TTVI is also associated with a slight but significant increase in transvalvular gradient (Figure 3).

The management of severe TR has gained momentum in recent years. It has been well established that severe TR is associated with high mortality and progression to end-stage right heart failure (2). Current guidelines recommend medical therapy with a focus on diuretics and treatment of associated left-sided conditions, pulmonary hypertension, and atrial fibrillation (1, 5). However, there is no direct evidence that medical therapy improves the dismal prognosis of severe TR. Isolated surgical treatment of isolated TR is relatively uncommon (40) and has a high in-hospital surgical mortality of 8–10% (6, 7, 41). This has been attributed to late referral and advanced comorbidities (7, 42). Recent data show a much lower surgical mortality (3.2%, 30-day mortality) in a Comprehensive Valve Center, which is likely due to patient selection (younger), preoptimization protocols, and surgical advances/expertise (43). The development of a less invasive catheter-based therapy is of high clinical relevance in this context. Several TTVI devices have been developed in recent years with various mechanisms of action and therapeutic targets. The initial TTVI experience showed that most procedures were well tolerated, with high procedural success and low in-hospital and early mortality. In this meta-analysis, we investigated the clinical and echocardiographic outcomes of multiple TTVI devices available on the market. We found that all-cause mortality at 1 year (25%, 95% CI: 15 to 37%) was substantially better than those reported in medically managed TR (36–46%) (40, 44, 45). However, the CIs overlap, and there is likely selection bias favoring patients who undergo TTVI. Thus, whether TTVI is superior to medical therapy for the management of severe TR remains a hypothesis that is currently being tested in several ongoing randomized clinical trials.

Surgical outcomes of TV repair/replacement are in the 3 to 10% range for in-hospital mortality and 10 to 30% at 1 year (46, 47), which is similar to the outcomes obtained by TTVI. Again, selection bias makes direct comparison challenging, but the results of TTVI are favorable and suggest that it is a reasonable option for patients at higher risk for surgery due to advanced age and comorbidities. A key finding of this meta-analysis is that procedural success offers a substantial survival gain compared with non-success. Achieving a TR grade ≤ 2+, together with an adequate device position and without intraoperative mortality, is associated with a 58% risk reduction in 1-year all-cause mortality. This suggests that TR reduction itself is the mechanism of benefit, similar to recent findings from a randomized clinical trial of TEER in functional mitral regurgitation (48). We did not find significant differences in the outcomes by the type of device, except for unsurprisingly higher mortality rates for heterotopic valve implantation of a commercially available device into the inferior vena cava (21). In the pathological cascade of TV disease, CAVI aims at the resolution of caval backflow that occurs at a late stage of severe TR. In our meta-analysis, only two studies reported data on this type of intervention, thus it is not possible to draw a definite conclusion (21, 28); however, the CAVI trial in Europe was stopped for safety reasons (21) and a small recent registry reported that 30-day mortality was estimated at 25% (49). Whether dedicated caval devices will have better outcomes awaits the results of ongoing trials (50, 51). We are also unable to detect differences between different devices relative to anatomic considerations. As the field of TTVI develops, it is increasingly recognized that certain anatomies may favor replacement devices over edge-to-edge repair or annuloplasty (52). For example, severe tethering of the leaflets into the RV or pacemaker-lead induced TR may favor replacement over repair, as is the case in surgery (53). Continued experience in device/patient selection may improve procedural success over time.

Overall, successful TTVI is associated with a significant reduction in TR severity, with complete elimination of TR in many cases (mostly TTVR). This was associated with reasonable mortality early and at 1 year. TTVI is associated with mild worsening of RV systolic function that is consistently observed within 30 days after a TTVI procedure and may represent preexisting mechanical dysfunction that is masked by the afterload reduction associated with TR. Whether this decline in RV function recovers over time could not be assessed in this meta-analysis. Our data show that despite a worsening in systolic function, importantly, FSV improves, probably contributing to the observed survival benefit, as also suggested by other investigators (37). Of note, the small but statistically significant increase in FSV (by continuity equation) might be the direct result of the important reduction of TR regurgitant flow. In addition, there are signs of reverse remodeling, evidenced by a reduction in the RV basal diameter and annulus dimensions at 30-day follow-up. Our meta-regression analysis showed that studies with larger mean tricuspid annulus diameter at baseline displayed higher 1-year mortality, thus highlighting the importance of timing in TTVI as well as optimal patient selection. The inverse relationship that we found between 1-year mortality and TAPSE appears counterintuitive at first glance. However, it must be noted that the range of TAPSE values was very limited (14.4–16.6 mm), thus one wonders how clinically relevant can this observation be. We were not able to assess RV-pulmonary artery coupling, which has been proposed as a superior measure of RV systolic function because it incorporates a measure of afterload (54). Additionally, how the reduction of the regurgitant flow impacts outcomes still remains to be investigated, as it could be an important determinant of survival.

### Limitations

This study suffers from the intrinsic limitations of all meta-analyses, particularly the selection bias relative to the non-randomized nature of the included studies. However, the use of multiple sensitivity analysis, as well as methods to assess study quality strengthens the power of the results. Additionally, we performed a meta-regression analysis to account for the high heterogeneity in some of our primary analyses. The fact that we only found 2 significant predictors of 1-year mortality suggests that despite a large number of included studies, baseline characteristics did not have a significant impact on our results.

## Conclusion

TTVI is safe and effective in reducing TR and may offer a survival advantage, although this will require confirmation in randomized clinical trials.

## Impact on Daily Life

Although a comparison to medical therapy is not yet available, transcatheter tricuspid interventions are effective in reducing TR severity and determining right ventricle reverse remodeling, in patients with severe TR, therefore emerging as a very promising alternative to conventional surgery.

## Data Availability Statement

The original contributions presented in the study are included in the article/Supplementary Material, further inquiries can be directed to the corresponding author/s.

## Author Contributions

AS and FI contributed to the conception, design of this study, and drafted the manuscript. PG contributed to the analysis and interpretation of the data for this study. RH, PLa, RS, PLu, GE, and PG critically revised the manuscript. All authors gave final approval and agree to be accountable for all aspects of the work ensuring integrity and accuracy.

## Conflict of Interest

AS receives grant support from Cardiovalve, Edwards Lifesciences, and W. L. Gore. PG receives grant support from Abbott Vascular, Medtronic, Boston-Scientific, Cardiovalve, Edwards, W.L. Gore, Medtronic, NeoChord and consulting fees from Abbott Vascular, Edwards, W. L. Gore, and 4C Medical. RH reports speaker fees from Abbott Structural, Baylis Medical, and Edwards Lifesciences; institutional educational and consulting contracts for which she receives no direct compensation with Abbott Structural, Boston Scientific, Edwards Lifesciences, Medtronic; equity with Navigate; and is Chief Scientific Officer for the Echocardiography Core Laboratory at the Cardiovascular Research Foundation for multiple industry-sponsored trials, for which she receives no direct industry compensation. PLu received grants from Abbott Vascular, Edwards Lifesciences, and ReCord. The remaining authors declare that the research was conducted in the absence of any commercial or financial relationships that could be construed as a potential conflict of interest.

## Publisher's Note

All claims expressed in this article are solely those of the authors and do not necessarily represent those of their affiliated organizations, or those of the publisher, the editors and the reviewers. Any product that may be evaluated in this article, or claim that may be made by its manufacturer, is not guaranteed or endorsed by the publisher.
