# Supplementary material for: Clinical and Echocardiographic Outcomes of Transcatheter Tricuspid Valve Interventions: A Systematic Review and Meta-Analysis
Source: Front Cardiovasc Med. 2022 Jul 11;9:919395. doi: 10.3389/fcvm.2022.919395 (PMC9309386; doi:10.3389/fcvm.2022.919395)
Supplement: Supplementary file 1 [file Table_1.DOCX]

Supplementary Material

# Supplementary Tables

# Supplementary Table 1. Newcastle-Ottawa quality assessment scale.

| **Author, Ref ^#^** | **Selection** | | **Comparability** | | **Outcome** | |
| --- | --- | --- | --- | --- | --- | --- |
|  | **Points (max 4)** | **Risk of bias** | **Points (max 2)** | **Risk of bias** | **Points (max 3)** | **Risk of bias** |
| **Ali et al. 2020** | 4 | Low | 2 | Low | 2 | Medium |
| **Besler et al. 2018** | 3 | Medium | 1 | Medium | 3 | Low |
| **Braun et al. 2018** | 3 | Medium | 2 | Low | 2 | Medium |
| **Braun et al. 2019** | 3 | Medium | 1 | Medium | 2 | Medium |
| **Cai et al. 2020** | 4 | Low | 2 | Low | 3 | Low |
| **Camplelo-Parada et al. 2015** | 2 | Medium | 1 | Medium | 2 | Medium |
| **Davidson et al. 2021** | 3 | Medium | 1 | Medium | 2 | Medium |
| **Dregrer et al. 2020** | 4 | Low | 2 | Low | 3 | Low |
| **Fam et al. 2019** | 2 | Medium | 1 | Medium | 2 | Medium |
| **Fam et al. 2021** | 2 | Medium | 1 | Medium | 2 | Medium |
| **Hahn et al. 2017** | 3 | Medium | 1 | Medium | 2 | Medium |
| **Hahn et al. 2020** | 2 | Medium | 1 | Medium | 2 | Medium |
| **Kitamura et al. 2021** | 2 | Medium | 1 | Medium | 3 | Low |
| **Kodali et al. 2021** | 3 | Medium | 1 | Medium | 2 | Medium |
| **Lurz et al. 2018** | 4 | Low | 2 | Low | 2 | Medium |
| **Lauten et al. 2018** | 2 | Medium | 1 | Medium | 2 | Medium |
| **Mehr et al. 2019** | 2 | Medium | 1 | Medium | 3 | Low |
| **Nickening et al. 2017** | 2 | Medium | 1 | Medium | 2 | Medium |
| **Nickening et al. 2019** | 3 | Medium | 1 | Medium | 3 | Low |
| **Nickening et al. 2019** | 3 | Medium | 1 | Medium | 3 | Low |
| **Orban et al. 2020** | 3 | Medium | 2 | Low | 3 | Low |
| **Perlman et al. 2018** | 2 | Medium | 2 | Low | 2 | Medium |
| **Perlman et al. 2017** | 3 | Medium | 1 | Medium | 3 | Low |
| **Rommel et al. 2019** | 3 | Medium | 1 | Medium | 3 | Low |
| **Ruf et al. 2021** | 3 | Medium | 1 | Medium | 2 | Medium |
| **Sugiura et al. 2020** | 3 | Medium | 2 | Low | 2 | Low |

Two independent reviewers undertook quality assessment and allocated stars/points for adherence to following criteria:

• Selection (adequate selection and definition of groups)

• Comparability (comparability of two groups for a selected variable and comparability for other variables)

• Outcome (modality of assessment, enough length of follow-up and adequacy of follow-up)

Studies with 4 stars for selection, 2 for comparability, and 3 for outcome were defined at low risk of bias. Studies with 2 or 3 stars for selection, 1 for comparability, and 2 for outcome were defined at medium risk. Any study with a score of 1 for selection or outcome ascertainment, or 0 for any of the three domains, was deemed at high risk of bias.

# Supplementary Table 2. Meta-regression analysis.

|  | **30-day mortality** | | **1-year mortality** | |
| --- | --- | --- | --- | --- |
|  | **p** | **slope** | **p** | **slope** |
| Atrial Fibrillation | 0.993 | 0.00 | 0.735 | -0.02 |
| Age | 0.349 | -0.10 | 0.227 | -0.44 |
| BMI | 0.662 | -0.38 | 0.205 | -1.83 |
| Concomitant MR treatment | 0.357 | -0.01 | 0.732 | -0.01 |
| Follow-up duration, weeks | 0.683 | 0.00 | 0.377 | 0.04 |
| Hypertension, % | 0.841 | 0.00 | 0.956 | 0.01 |
| LVEF, % | 0.223 | -0.06 | 0.429 | -0.11 |
| Male, % | 0.739 | 0.01 | 0.882 | -0.01 |
| N patients | 0.156 | -0.01 | 0.419 | -0.01 |
| NYHA class III-IV, % | 0.424 | 0.02 | 0.290 | 0.07 |
| PASP, mean | 0.727 | -0.01 | 0.732 | 0.11 |
| Previous CABG, % | 0.066 | 0.05 | NA | NA |
| Previous Cardiac Surgery, % | 0.244 | 0.01 | 0.507 | 0.01 |
| Previous PMK lead, % | 0.328 | 0.02 | 0.101 | 0.13 |
| Previous AVR, % | 0.081 | 0.04 | NA | NA |
| Previous MVR, % | 0.780 | 0.01 | NA | NA |
| RA Volume, mean | 0.801 | 0.01 | 0.072 | -0.05 |
| RV FAC, % | 0.471 | -0.07 | NA | NA |
| RV basal diameter, mean | 0.258 | 0.02 | 0.052 | NA |
| TAPSE, mean | 0.569 | -0.09 | **0.029** | **1.29** |
| TR EROA, mean | 0.144 | 0.97 | 0.338 | 2.48 |
| TR Reg Volume, mean | 0.503 | 0.01 | NA | NA |
| Tricuspid annulus diameter, mean | 0.758 | 0.02 | **0.040** | **0.42** |

## Supplementary Figures

Full-text articles excluded, with reasons (n = 11):

- Duplicate data (n = 6)
- Study exploring different outcomes (n = 5)
- Design studies (n = 1)

Records identified through other sources
(n = 0)

Records identified through database searching
(n = 2718)

Records after duplicates removed
(n = 720)

Records excluded
(n = 1959)

Studies included in quantitative synthesis (meta-analysis)
(n = 27)

Studies included in qualitative synthesis
(n = 27)

Full-text articles assessed for eligibility
(n = 39)

Records screened
(n = 1998)

Identification

Eligibility

Included

Screening

**Supplementary Figure 1.** Meta-analysis flow-chart.

**
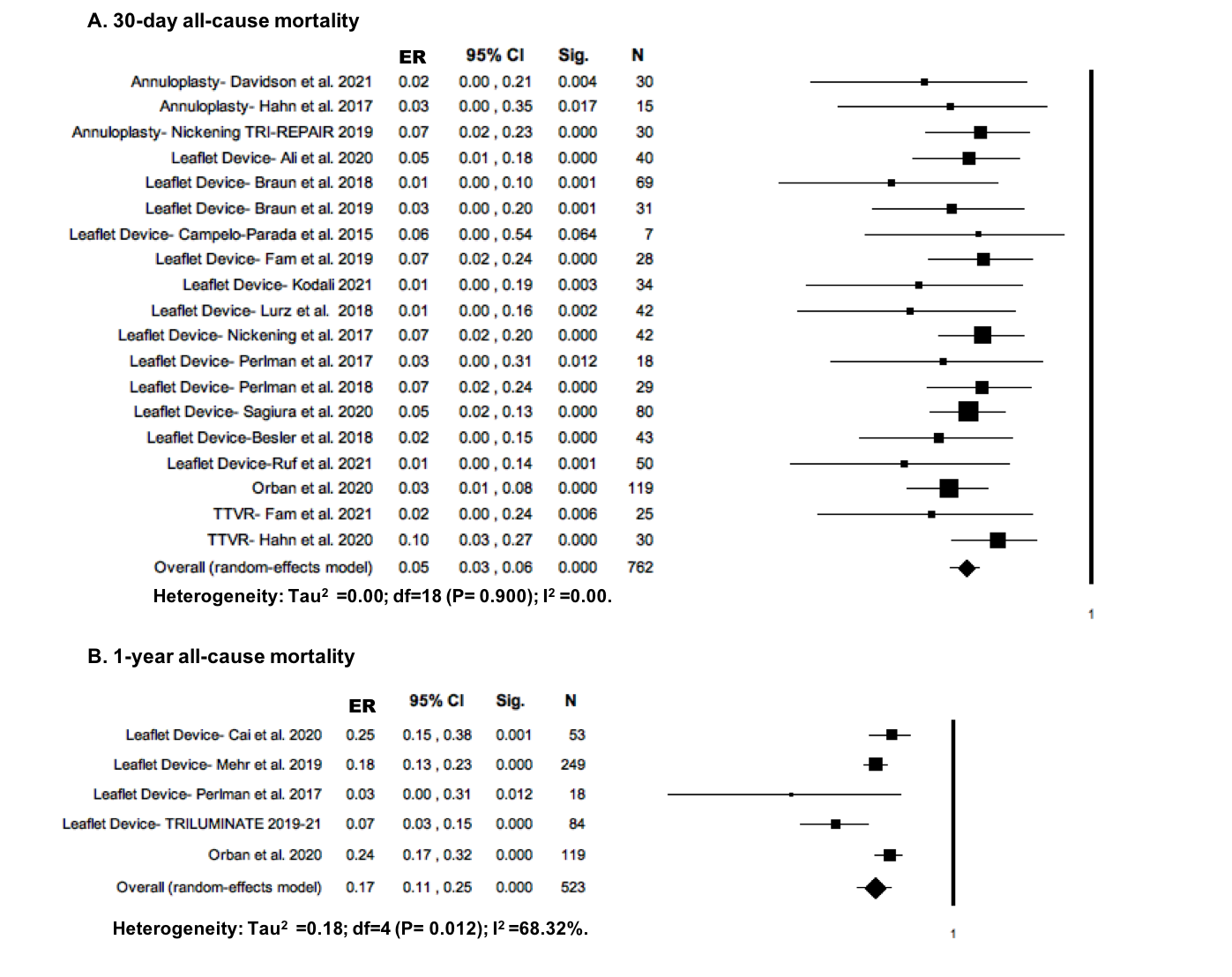
**

**Supplementary Figure 2.** Sensitivity analyses for 30-day (A) and 1-year mortality (B), after removing CAVI studies.

**
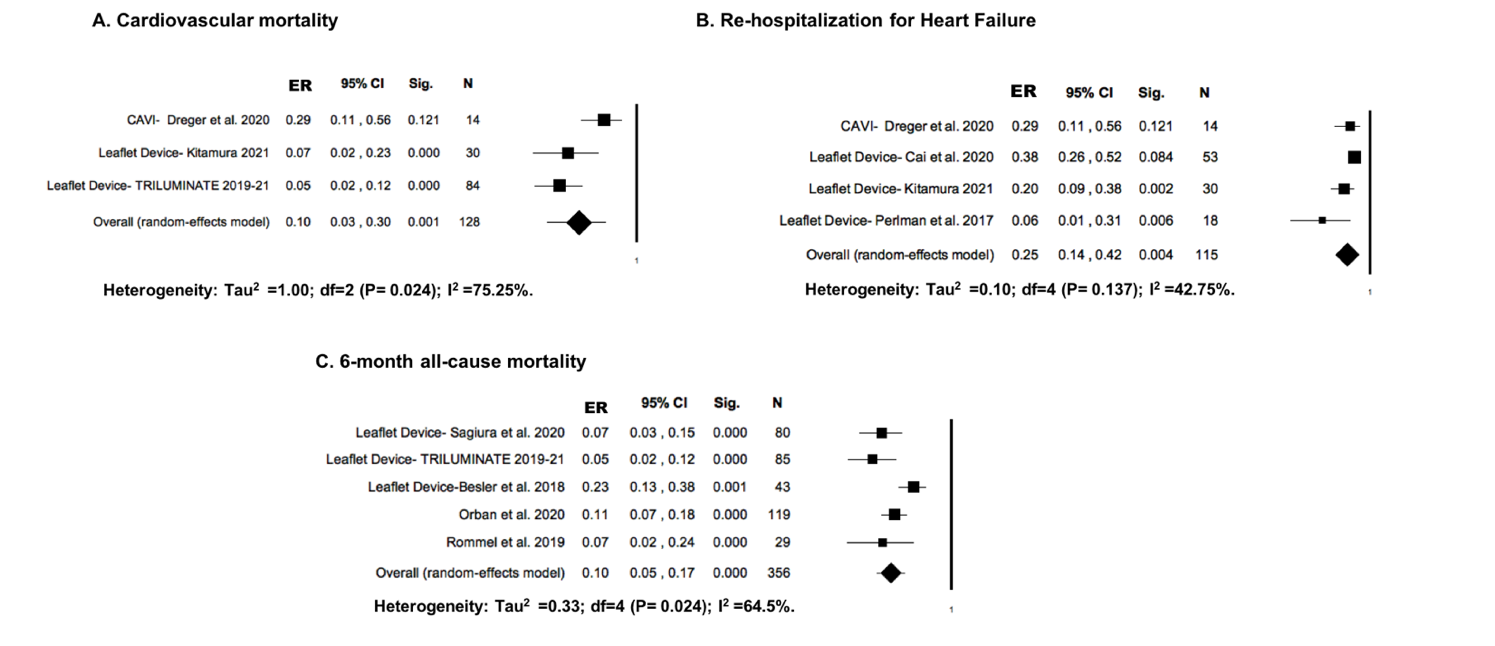
**

**Supplementary Figure 3.** Forrest-plot for the incidence of 1-year cardiovascular mortality (A), re-hospitalization for heart failure (B), and 6-month all-cause mortality.


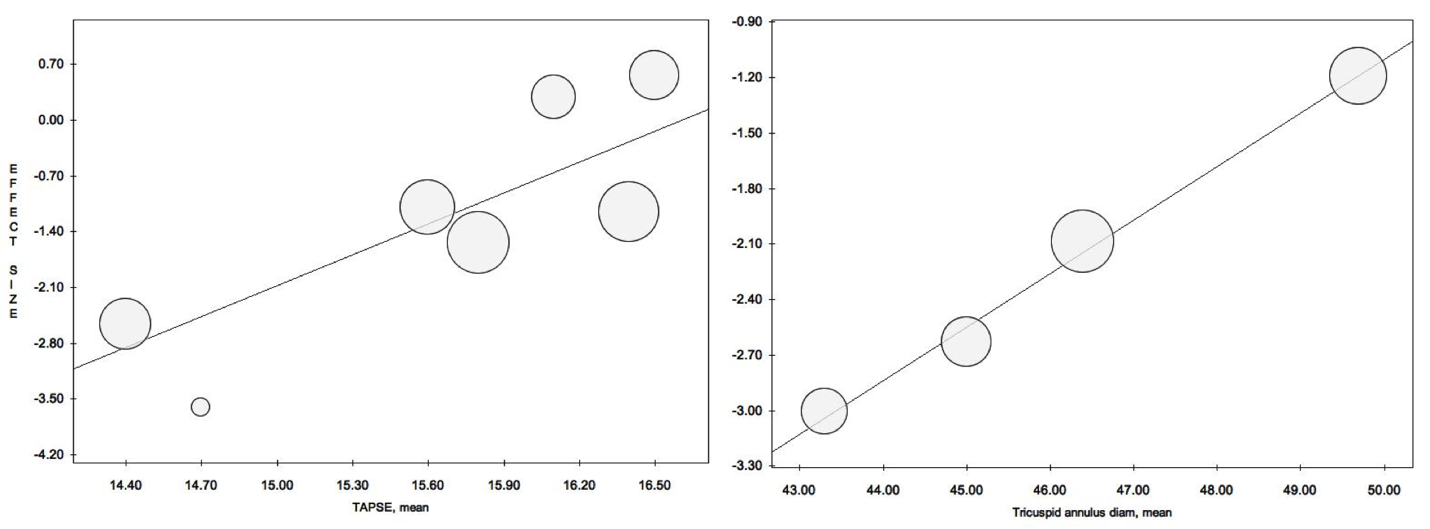


**Supplementary Figure 4.** Representative plots of significant effects modifiers.


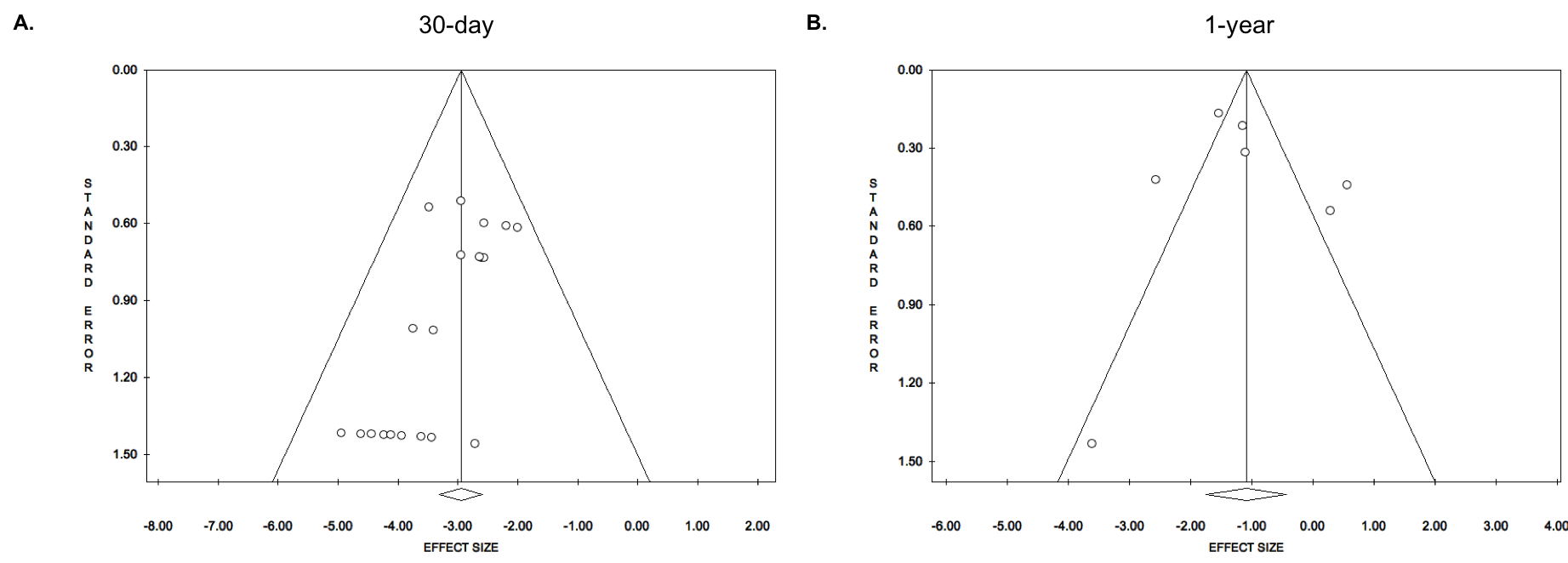


**Supplementary Figure 5.** Representative funnel plots for the analyzed outcomes of interest.
